# Supplementary material for: SPF: A spatial and functional data analytic approach to cell imaging data
Source: PLoS Comput Biol. 2022 Jun 15;18(6):e1009486. doi: 10.1371/journal.pcbi.1009486 (PMC9239468; doi:10.1371/journal.pcbi.1009486)
Supplement: S1 Text — (PDF) [file pcbi.1009486.s001.pdf]

Supporting Information for "SPF: A Spatial and Functional Data Analytic Approach to cell Imaging data"

Thao Vu[1], Julia Wrobel[1], Benjamin G. Bitler [2,3], Erin L. Schenk[4], Kimberly R. Jordan [5], Debashis Ghosh[1*]

1 Department of Biostatistics and Informatics, University of Colorado Anschutz Medical Campus, Aurora, CO 80045, USA

2 University of Colorado Comprehensive Cancer Center, Aurora, CO 80045, USA

3 Department of OB/GYN, Division of Reproductive Sciences, The University of Colorado, Aurora, CO 80045, USA

4 Department of Medicine, University of Colorado Anschutz Medical Campus, Aurora, CO 80045, USA

5 Department of Immunology and Microbiology, University of Colorado School of Medicine, Aurora, CO 80045, USA

*debashis.ghosh@cuanschutz.edu

S.1 NSCLC dataset

S.1.1 Model output by fitting mcf

```
Family: Cox PH
Link function: identity


Formula:
stime ~ stage_group + Age + total_cell + ti(t_int, func_cov,
    by = l_int, bs = c("cr", "cr"), k = c(k, k), mc = c(FALSE,
        TRUE))


Parametric Terms:
```

```
             df Chi.sq  p-value
stage_group  3   6.938 0.073913
Age          1  13.574 0.000229
total_cell   1   1.940 0.163675

Approximate significance of smooth terms:
                             edf Ref.df  Chi.sq p-value
ti(t_int,func_cov):l_int 7.164   9.430   8.871   0.442
```

S.1.2 Exploratory analysis of MHCII in tumor vs. stromal cells

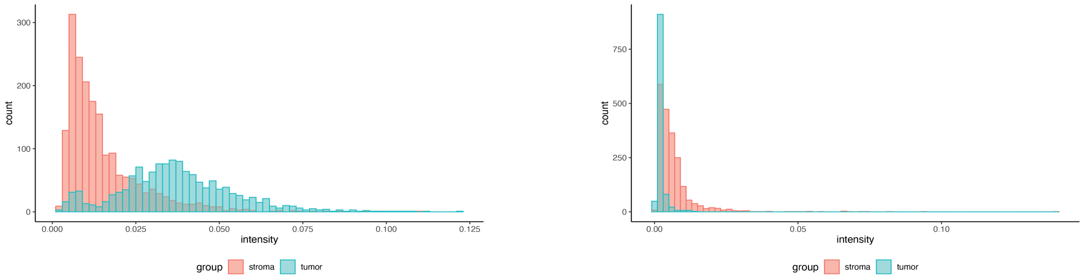

(a) A representative MHCII^{hi} sample

(b) A representative MHCII^{lo} sample

Fig S1. Distribution of MHCII expression in tumor (turquoise) vs. stromal (red) cells in (A) A representative MHCII^{hi} sample. (B) A representative MHCII^{lo} sample.

S.1.3 Model output by fitting Moran's I correlation in MHCII

```
Family: Cox PH
Link function: identity


Formula:
stime ~ stage_group + Age + total_cell + ti(t_int, func_cov,
    by = l_int, bs = c("cr", "cr"), k = c(k, k), mc = c(FALSE,
        TRUE))
```

Parametric Terms:

	df	Chi.sq	p-value
stage_group	3	6.058	0.108807
Age	1	13.326	0.000262
total_cell	1	1.264	0.260872

Approximate significance of smooth terms:

	edf	Ref.df	Chi.sq	p-value
ti(t_int,func_cov):l_int	2.349	2.584	0.449	0.873

S.2 TNBC dataset

S.2.1 Representative TNBC images

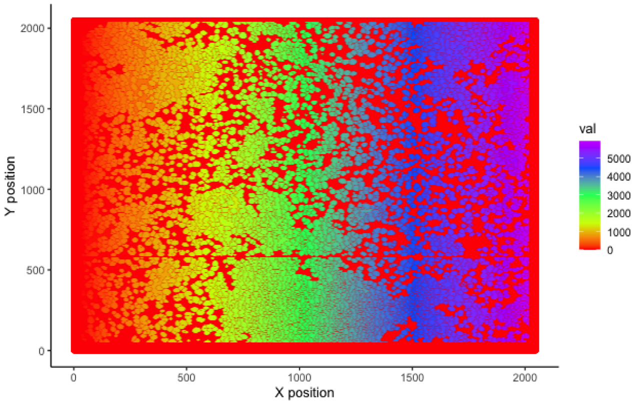

(a) Pixel-level image for Patient 1

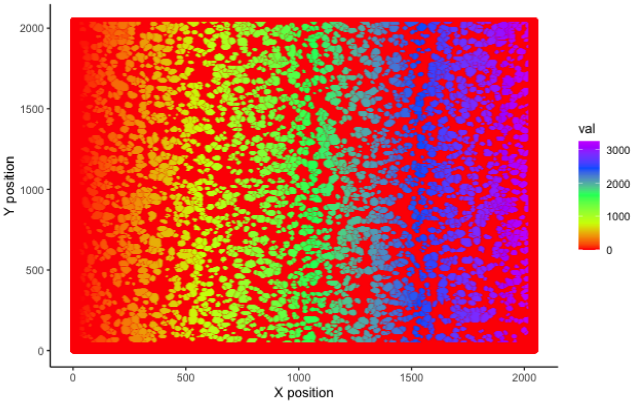

(b) Pixel-level image for Patient 2

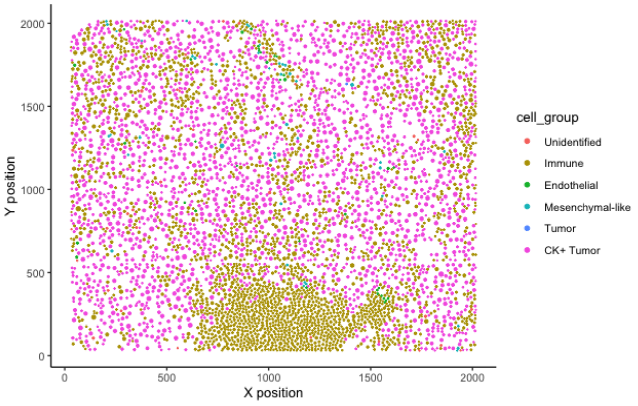

(c) Cell-level image for Patient 1

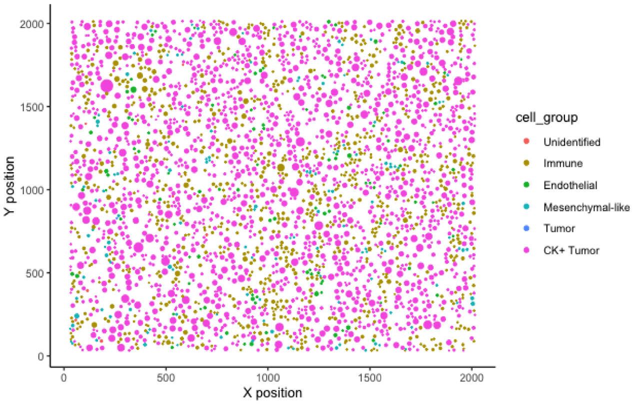

(d) Cell-level image for Patient 2

Fig S2. Representative TNBC tissue samples. Top Row: Pixel-level images of (A) Patient 1 and (B) Patient 2; color-coded from cell segmentation process to be associated with cell-level data. Bottom Row: Corresponding cell-level images for (C) Patient 1 and (D) Patient 2. Each color represents a cell classification group. Dot size is proportional to cell size.

S.2.2 Exploratory analysis of P53 in tumor vs. immune cells

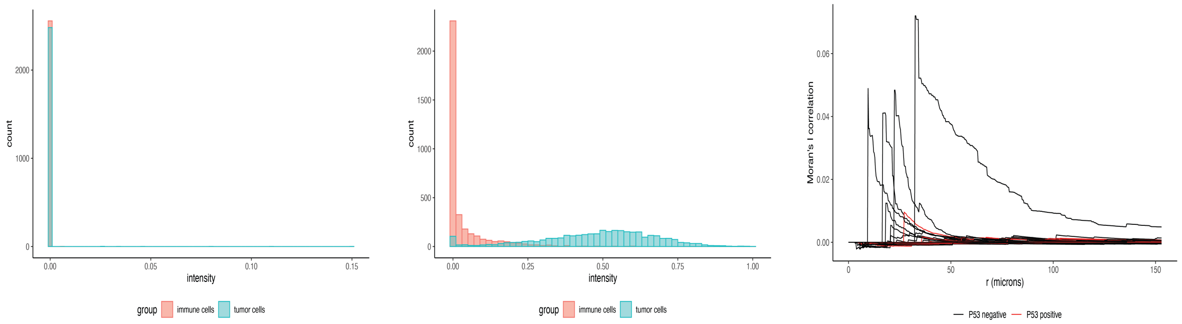

(a) A representative P53 positive sample

(b) A representative P53 negative sample

(c) Moran's I correlation using P53 expression

Fig S3. P53 expression in TNBC. Distribution of P53 expression in tumor (turquoise) vs. immune cells (red) in: (A) A representative P53 positive sample. (B) A representative P53 negative sample. (C) Corresponding Moran's I correlation using P53 expression in P53 positive (red) and P53 negative (black) samples. Moran's I values above 0 indicate a direct relationship in P53 expression between tumor and immune cells. Negative Moran's I values suggest an inverse association in P53 expression between tumor and immune cells.

S.3 Ovarian cancer dataset

Tissue microarray (TMA) slides of 132 ovarian cancer patients were stained with antibodies specific for CD3, CD4, CD8, CD19, CD68, cytokeratin, Ki67, pStat, and IER3. The slides were imaged using Vectra 3.0 microscope (Akoya Biosystems) and then segmented to define cells within the tumor and stroma regions as well as to assign cell phenotype utilizing `inForm` software. More details can be found in Jordan et al. [1]. Fig. S4 visualized images of four representative patients with turquoise and red points denoting tumor and stromal cells, respectively. Within the cohort, we excluded 18 patients from the analysis due to missingness of clinical information. Similar to the applications described in the main text, we explored the impact of spatial distribution of cells in the TME on patient survival outcome. Fig. S5 (A) captured the interactions between tumor and stromal cells as a function of cell distance for all patients in the dataset while Fig. S5 (B) depicted the Moran's I correlation between tumor and stromal cells within the TME with respect to Ki67 marker expression. Mcf and Moran's I curves were separately included in the model as functional covariates with survival outcome displayed in Fig. S5 (C). The estimated functional surface shown Fig. S6 (A) had the values decreased from red to blue, in

correspondence with a decrease in hazard of mortality, while holding the scalar predictors fixed. If there were more stromal cells surrounding tumor cells at any given distance r, which resulted in the larger mcf values, the hazard of death increased. On the contrary, if tumor and stromal cells were clustered together in their compartments, which resulted in smaller mcf values, the estimated survival improved. In similar fashion, Fig. S6 (B) displayed the estimated functional surface $\hat{F}$ after fitting the model using the Moran's I correlation curve in Ki67 expression as functional covariates. Specifically, positive correlation in Ki67 intensity between tumor and stromal cells in the neighborhood of less than 150μm caused an increase in the risk of mortality. However, if the positive association continued past 175μm, the hazard of mortality decreased.

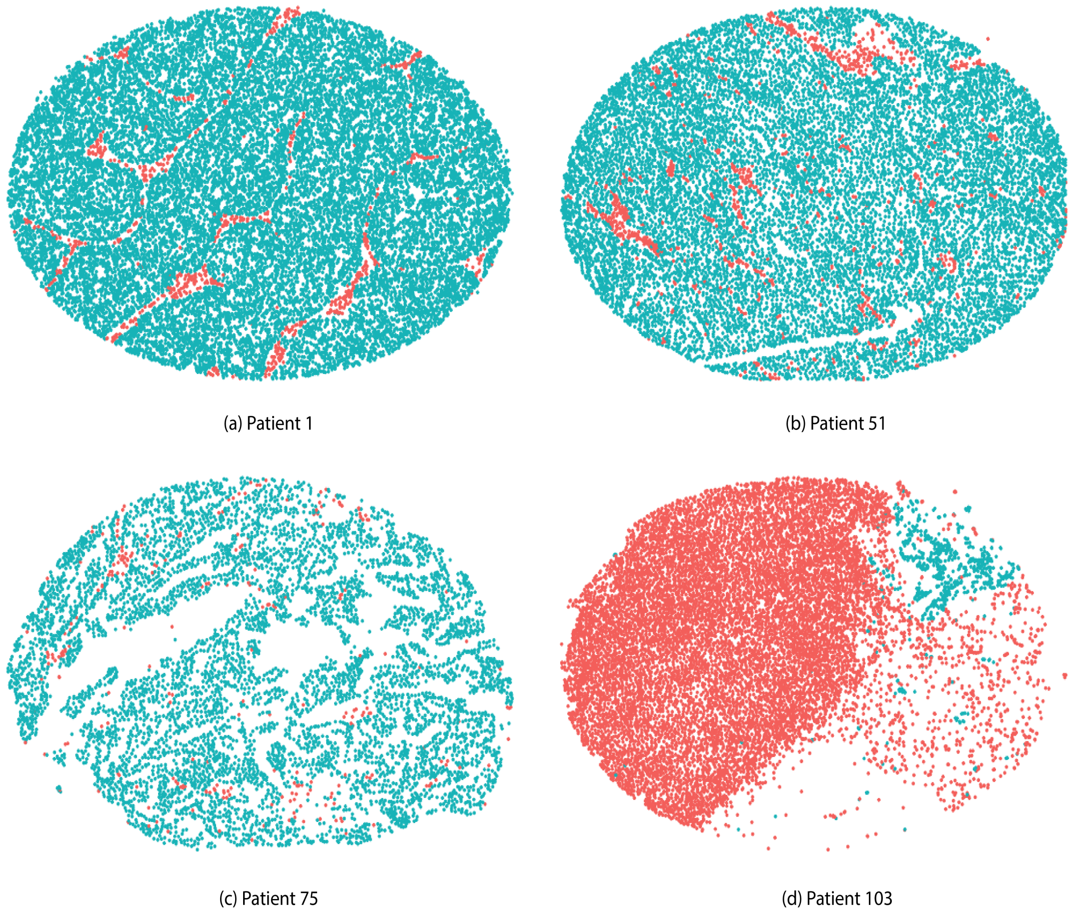

Fig S4. Representative images in the ovarian cancer dataset. Example images of four representative patients (A) Patient 1, (B) Patient 51, (C) Patient 75, and (D) Patient 103, with red and turquoise points denoting stromal and tumor cells, respectively.

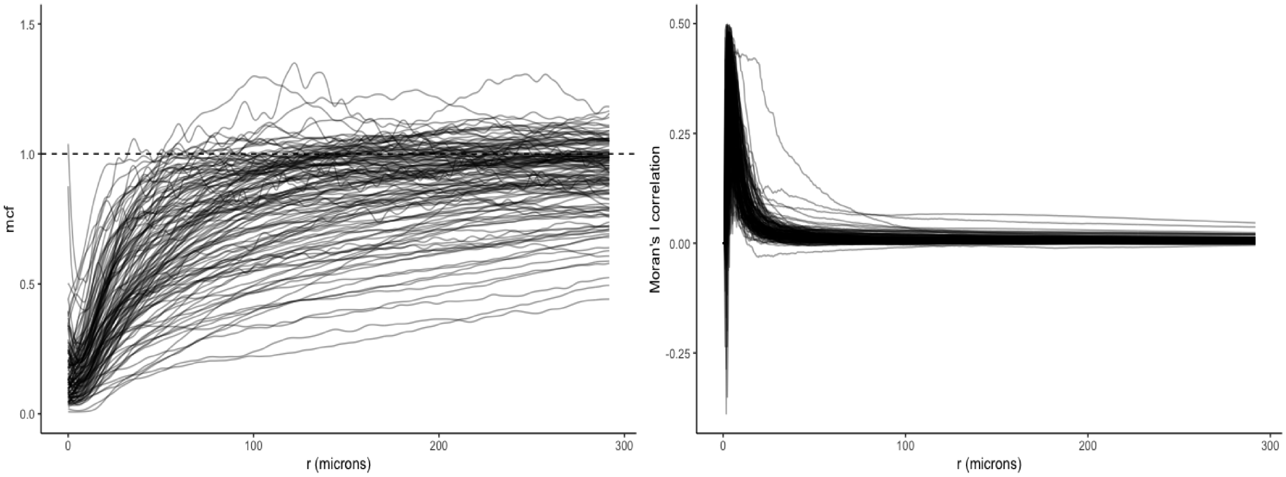

(a) All mcf's

(b) Moran's I correlation for Ki67

Fig S5. Ovarian cancer dataset. (A) Mcf curves for all patients. Note that mcf values below 1 indicate strong clustering of cells of same type, while values above 1 suggest higher level of mixing in of the two cell types. (B) Moran's I correlation between tumor and stromal cells across subjects using Ki67 marker expression. Moran's I values above 0 indicate a direct relationship in Ki67 expression between tumor and stromal cells. Negative Moran's I values suggest an inverse association in Ki67 expression between tumor and stromal cells.

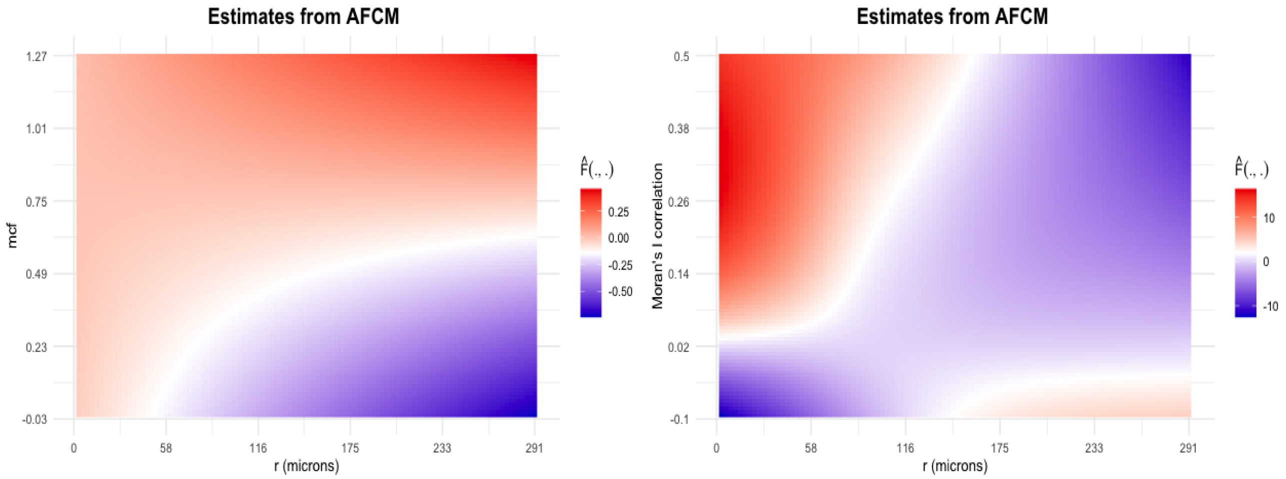

(a)AFCM using Mcf

(b) AFCM using Moran's I correlation in Ki67

Fig S6. Estimated surfaces from AFCM using ovarian cancer dataset. (A) Estimated surface from AFCM using mcf curves as functional covariates, with values of $\hat{F}$ decreasing from positive (red) to negative (blue). (B) Estimated surface from AFCM using Moran's I correlation in Ki67 expression between tumor and stromal cells, with values of $\hat{F}$ decreasing from positive (red) to negative (blue). Positive $\hat{F}$ corresponds to increased risk of mortality while negative $\hat{F}$ associates with reduced hazard of death.

References

1. Jordan KR, Sikora MJ, Slansky JE, Minic A, Richer JK, Moroney MR, et al. The capacity of the ovarian cancer tumor microenvironment to integrate inflammation signaling conveys a shorter disease-free interval. Clinical Cancer Research. 2020;26(23):6362–6373.
