# Supplementary figures and images for: SPF: A spatial and functional data analytic approach to cell imaging data
Source: PLoS Comput Biol. 2022 Jun 15;18(6):e1009486. doi: 10.1371/journal.pcbi.1009486 (PMC9239468; doi:10.1371/journal.pcbi.1009486)

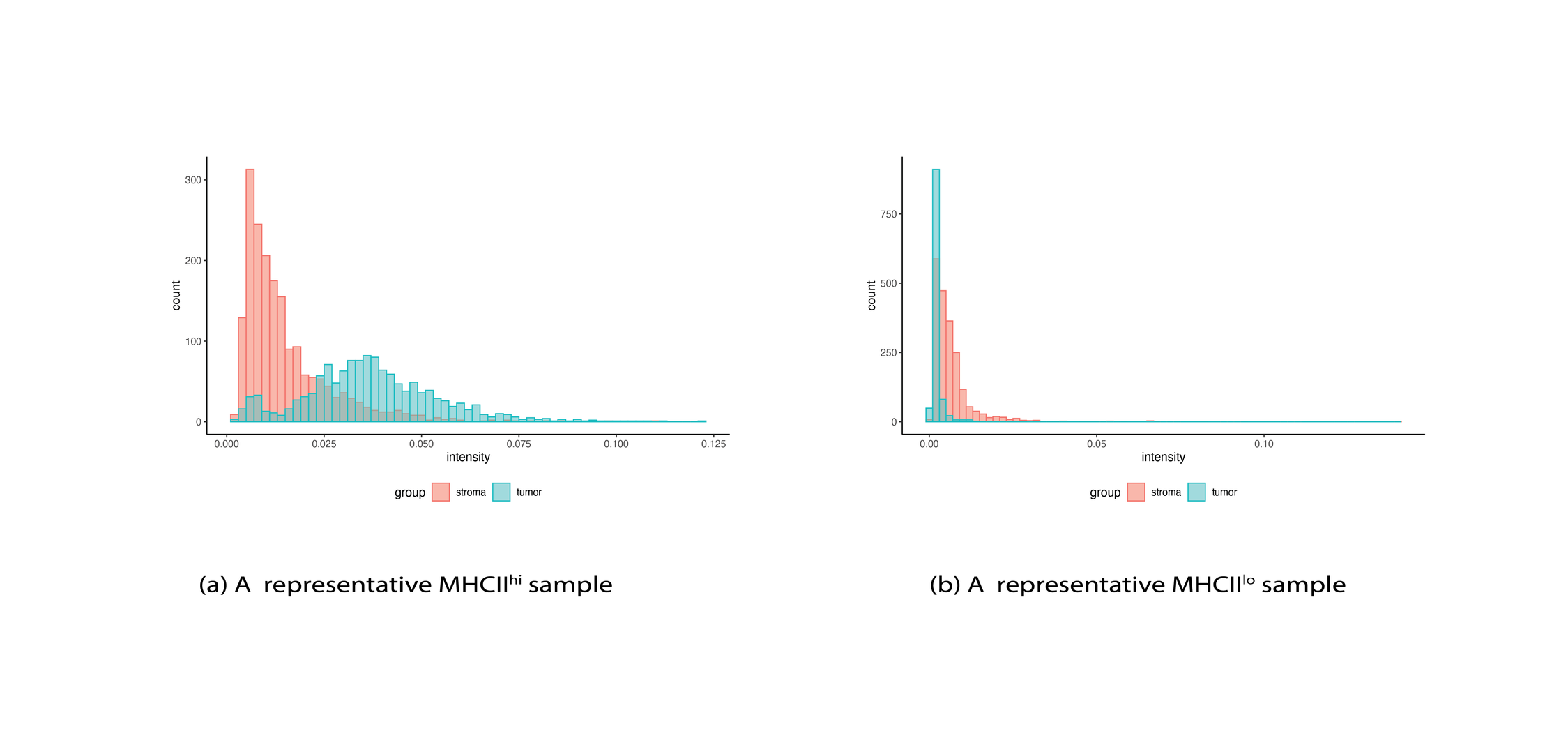

Supplement: S1 Fig — Distribution of MHCII expression in tumor (turquoise) vs. stromal (red) cells in (A) A representative MHCIIhi sample. (B) A representative MHCIIlo sample. (TIF) [file pcbi.1009486.s002.tif]

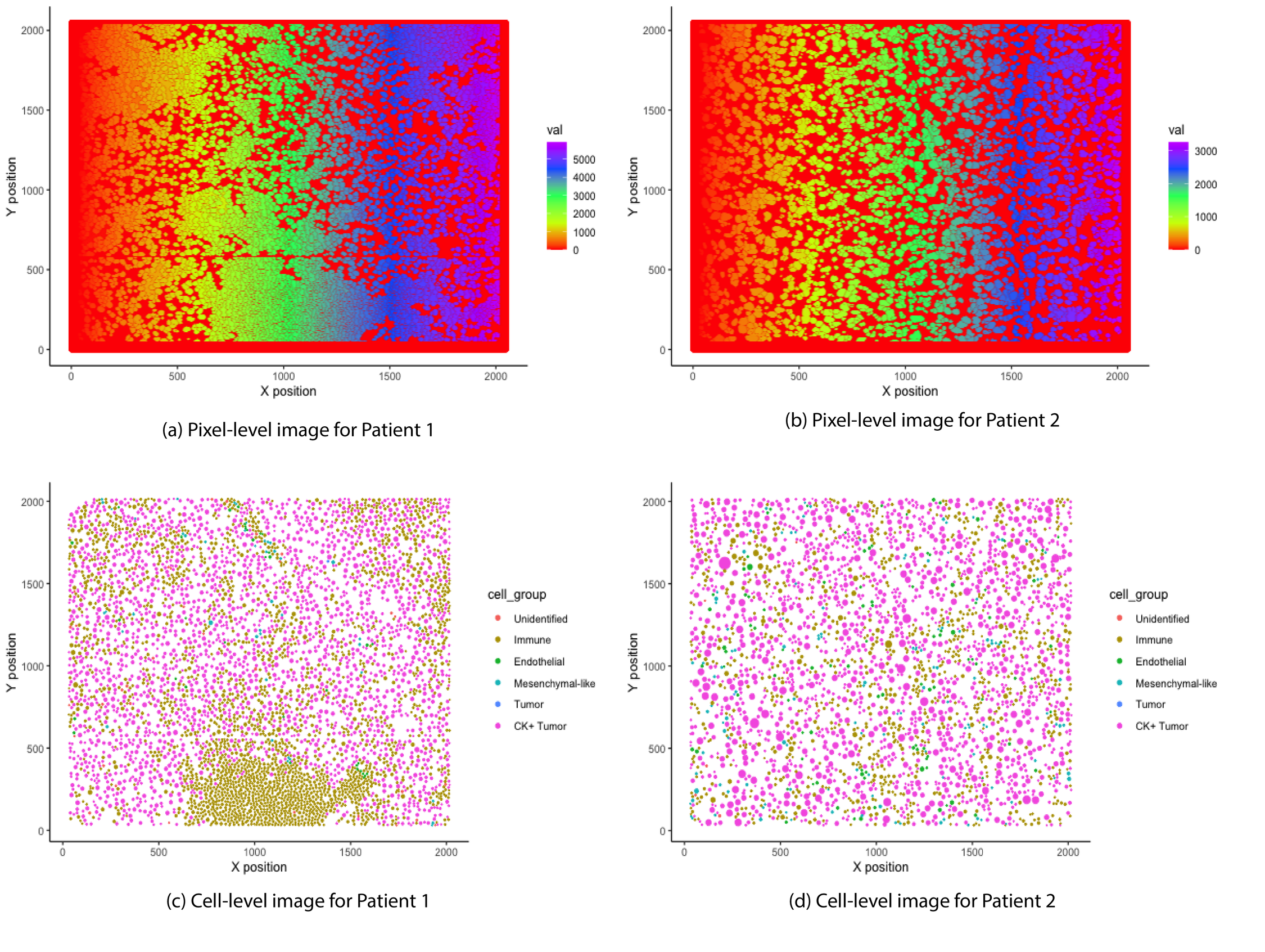

Supplement: S2 Fig — Top Row: Pixel-level images of (A) Patient 1 and (B) Patient 2; color-coded from cell segmentation process to be associated with cell-level data. Bottom Row: Corresponding cell-level images for (C) Patient 1 and (D) Patient 2. Each color represents a cell classification group. Dot size is proportional to cell size. (TIF) [file pcbi.1009486.s003.tif]

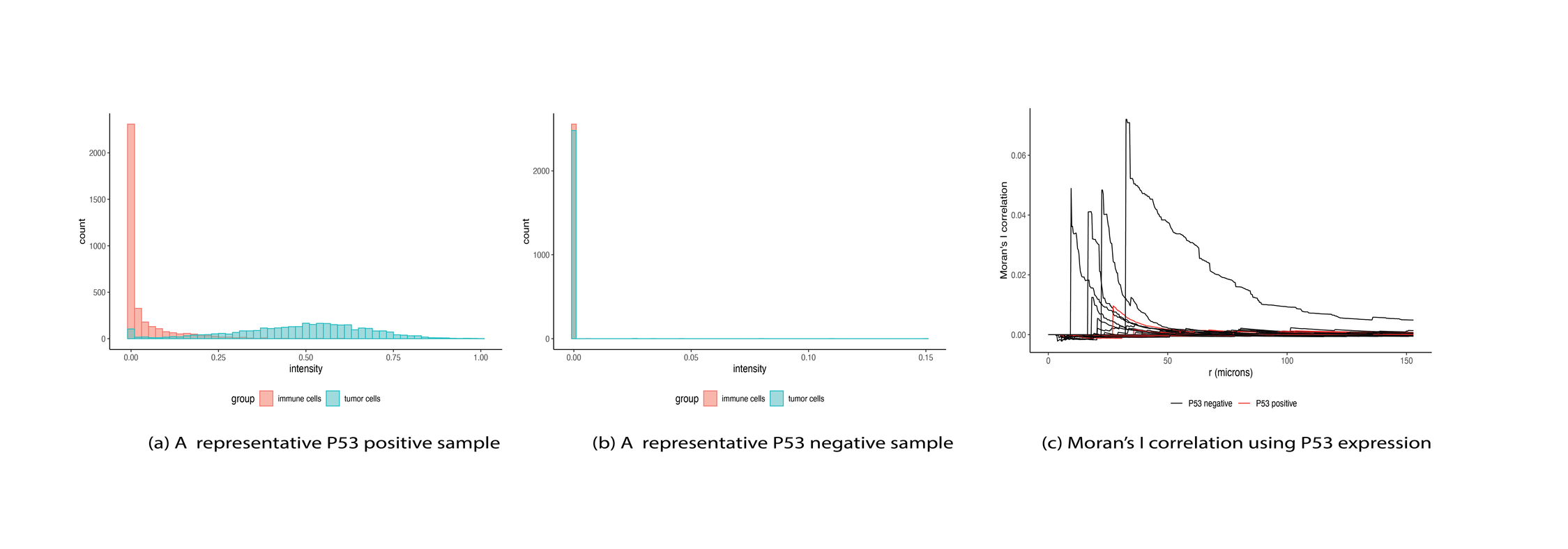

Supplement: S3 Fig — Distribution of P53 expression in tumor (turquoise) vs. immune cells (red) in: (A) A representative P53 positive sample. (B) A representative P53 negative sample. (C) Corresponding Moran’s I correlation using P53 expression in P53 positive (red) and P53 negative (black) samples. Moran’s I values above 0 indicate a direct relationship in P53 expression between tumor and immune cells. Negative Moran’s I values suggest an inverse association in P53 expression between tumor and immune cells. (TIF) [file pcbi.1009486.s004.tif]

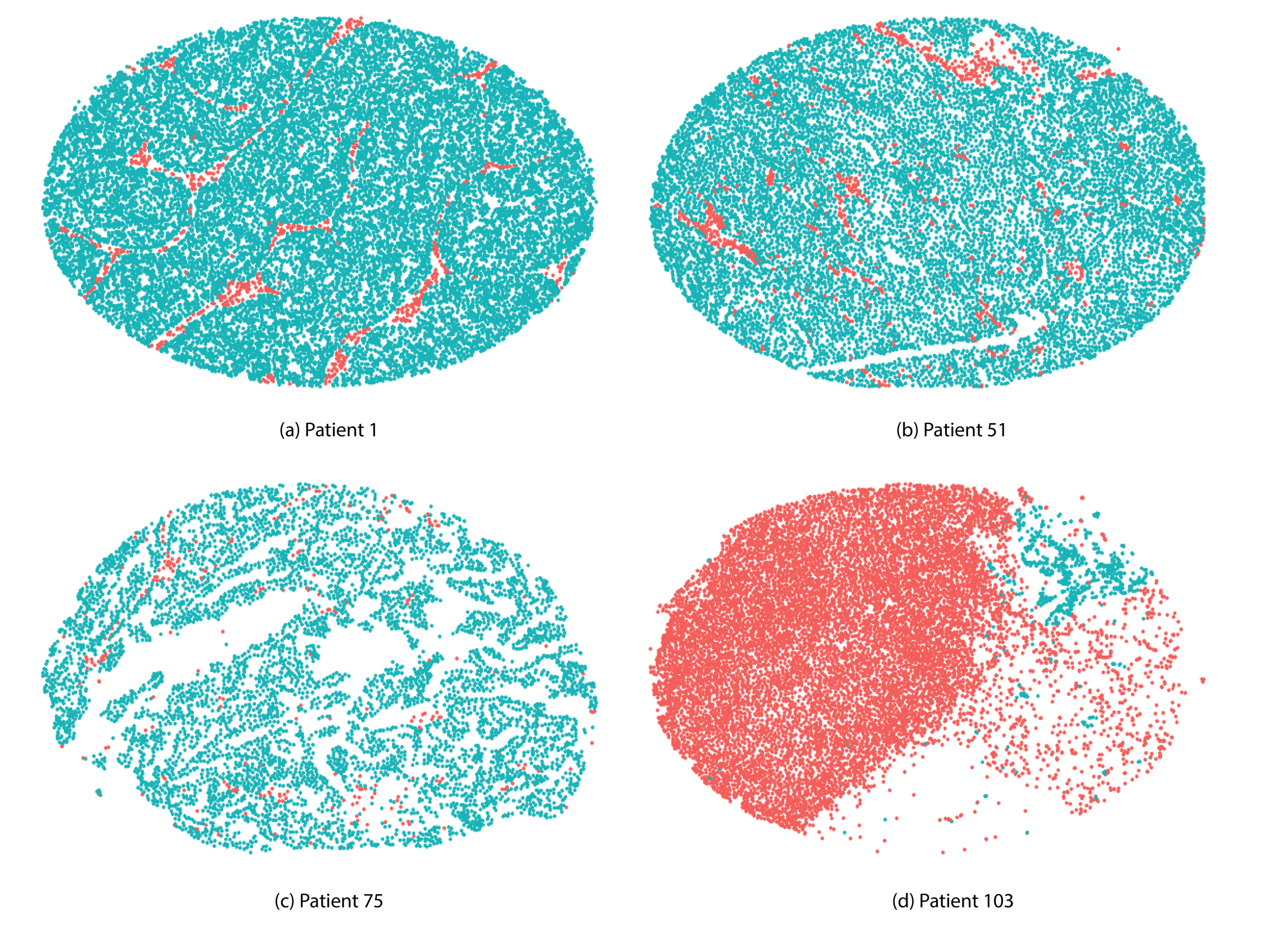

Supplement: S4 Fig — Example images of four representative patients (A) Patient 1, (B) Patient 51, (C) Patient 75, and (D) Patient 103, with red and turquoise points denoting stromal and tumor cells, respectively. (TIF) [file pcbi.1009486.s005.tif]

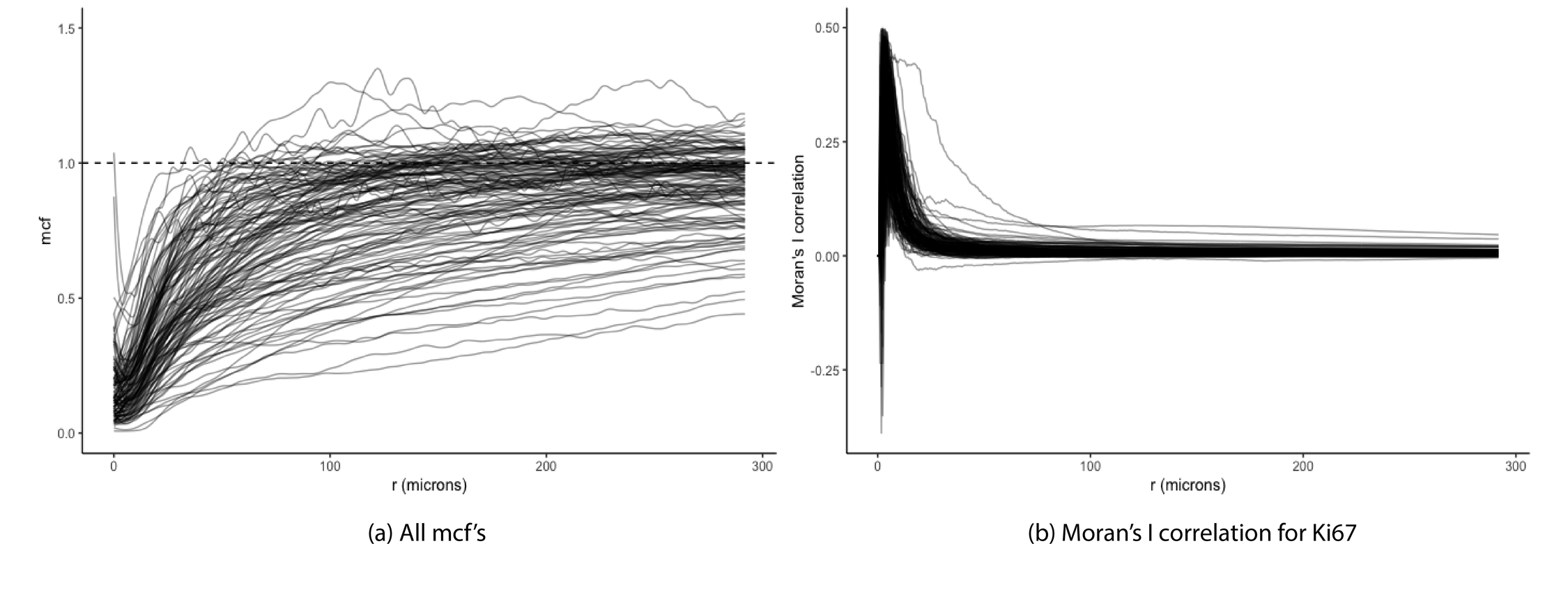

Supplement: S5 Fig — (A) Mcf curves for all patients. Note that mcf values below 1 indicate strong clustering of cells of same type, while values above 1 suggest higher level of mixing in of the two cell types. (B) Moran’s I correlation between tumor and stromal cells across subjects using Ki67 marker expression. Moran’s I values above 0 indicate a direct relationship in Ki67 expression between tumor and stromal cells. Negative Moran’s I values suggest an inverse association in Ki67 expression between tumor and stromal cells. (TIF) [file pcbi.1009486.s006.tif]

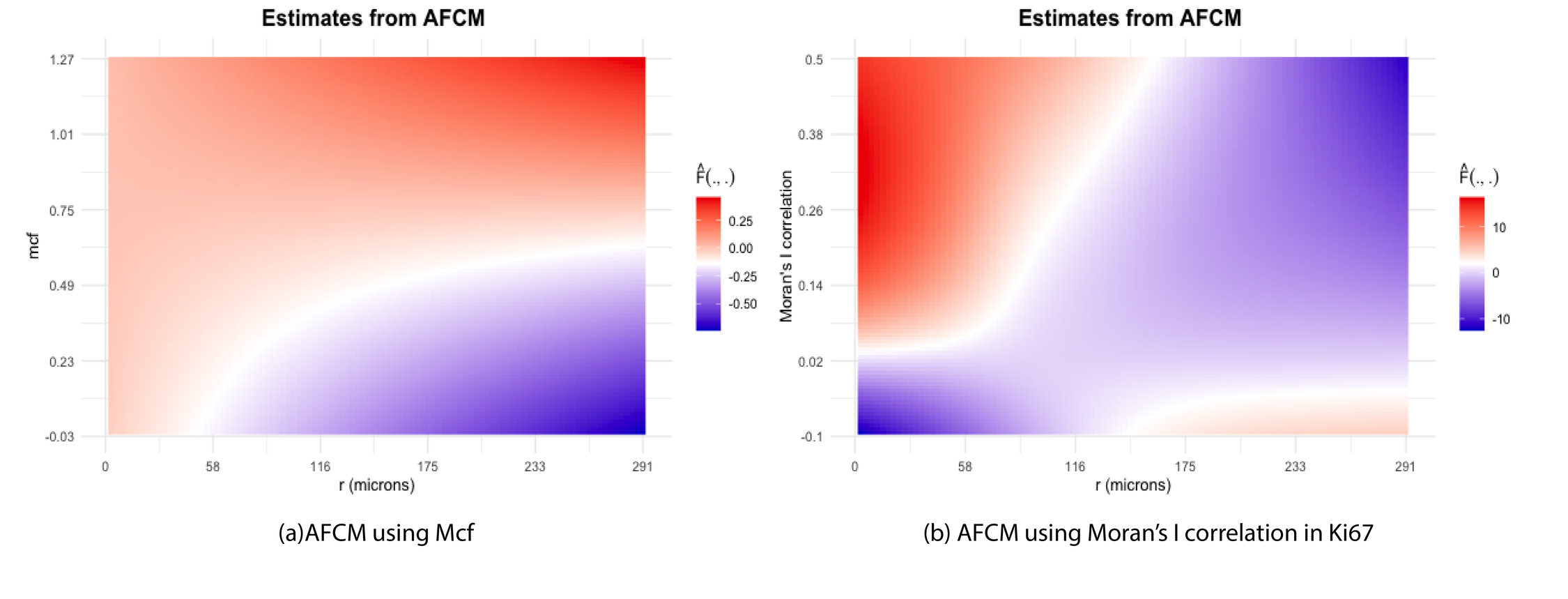

Supplement: S6 Fig — (A) Estimated surface from AFCM using mcf curves as functional covariates, with values of F^ decreasing from positive (red) to negative (blue). (B) Estimated surface from AFCM using Moran’s I correlation in Ki67 expression between tumor and stromal cells, with values of F^ decreasing from positive (red) to negative (blue). Positive F^ corresponds to increased risk of mortality while negative F^ associates with reduced hazard of death. (TIF) [file pcbi.1009486.s007.tif]
