# Supplementary material for: SPF: A spatial and functional data analytic approach to cell imaging data
Source: PLoS Comput Biol. 2022 Jun 15;18(6):e1009486. doi: 10.1371/journal.pcbi.1009486 (PMC9239468; doi:10.1371/journal.pcbi.1009486)
Supplement: Attachment — Submitted filename: ResponseLetter.pdf [file pcbi.1009486.s008.pdf]

Dear Dr. Meier-Schellersheim and Dr. Haugh,

Thank you very much for reviewing our manuscript entitled: "SPF: A Spatial and Functional Data Analytic Approach to cell Imaging data" (PCOMPBIOL-D-21-01681). We are pleased to submit our revised manuscript for publication in PLOS Computational Biology. Two versions of the revised paper are included: one with changes highlighted in yellow and a clean version.

We really appreciate your providing us constructive comments and suggestions, which led to a significant improvement of our paper. To facilitate the review process, in our letter, we provide a point-by-point response to each of the comments and mention the changes made to the original manuscript. To summarize, below are the highlights of major changes:

- We have reorganized the paper by moving the paragraphs describing the two datasets (NSCLC and TNBC) to the last part of the Materials and Methods Section.
- We have edited all the surface plots in Figures 4 and 6, such that the estimated values are transitioned from red (i.e., positive) to blue (i.e., negative), and centered on white (i.e., 0) to make the result interpretations easier. Furthermore, all figure legends have been expanded with more details.
- In the Application results Section, we have included the motivation for the analyses regarding the correlation in marker expression of MHCII in tumor vs. stromal cells (NSCLC) and of P53 in tumor vs. immune cells (TNBC).
- Lastly, in the Conclusions and Discussion Section, we have included possible generalizations of our proposed framework to investigation of spatial architecture of cells beyond the TME as well as response variables other than survival outcomes.

Thank you very much for your time and consideration. We look forward to hearing from you.

Sincerely,

Thao Vu, Ph.D.

Responses to Reviewer 1

We sincerely thank you for providing us constructive suggestions, which led to a significant improvement of the paper. We now provide point to point responses to your comments. For your convenience, we reiterate your comments in italics before responding.

1. *The authors have proposed a novel approach to modelling the spatial interactions between cells in the tumour microenvironment (TME) to identify patterns associated with patient survival. The effectiveness of this approach has been demonstrated on publicly available data. While the focus of the manuscript is on the TME, this approach is clearly generalisable to other contexts and, because of this, I will likely be integrating components of their analysis strategy into any future analysis I perform.*

   Our response: We thank you for your positive comment.

2. *Estimates from AFCM plots: Could you center all of the colours on zero? i.e. red positive and blue negative.*

   Our response: Thank you for your suggestion. We have updated the surface plots so that the color scale is transitioned from red (i.e., positive) to blue (i.e., negative), and centered on white (i.e., 0). Please see Figures 4 and 6 in our revised manuscript.

3. *Line 122: "Specifically, tumor cells and their immediate neighboring stromal cells are negatively correlated in MHCII expression when they are less than 10 microns apart. As the cross-type cell distance increases, the correlation rises at different rates, and eventually drops close to 0." Given the average diameter of a cell, what does MHCII becoming negatively correlated when cells are less than 10 microns mean? Are there worries about poor segmentation?*

   Our response: In response to your useful comment, we reinvestigated the distribution of the cross distances between cells in the tumor and stroma regions across 153 patients in the NSCLC dataset, as well as the distribution of cell diameters. It is worth pointing out that we used the state-of-the-art commercial software, inForm, for cell and nucleus segmentation. While it is possible that poor segmentation can occur with this software, this has not been thoroughly explored and is beyond the scope of our current analysis.

   We have added the following paragraph to the second paragraph of Section 3.1.

   "Note that in the 2D images captured by slicing 3D tissue samples, biological overlapping could potentially occur, however our 2D samples were sliced as thin as possible ($\approx 4\ \mu m$ per

section) to minimize this potential overlap. Additionally, by investigating the diameters of tumor and stromal cells in the dataset, we noticed that the median diameter was about 7.2 μm. Altogether, it was possible to observe pairs of cross-type cells in the neighborhood less than 10 μm. Moreover, the tumor and stromal cells in such small radius span tended to have an inverse relationship in MHCII expression, i.e., tumor cells with low MHCII were likely to be adjacent to high MHCII stromal cells, leading to negative Moran's I correlation as seen Fig. 2."

4. *It would be helpful if the figure legends were more verbose, informative and/or repeated some of the information in the text. For example "Fig 4. Estimated surface from AFCM using lung cancer dataset. (a) Mcf curves and (b) Moran's I correlation using MHCII expression". It would be very helpful for a reader (me) if you fleshed out how to interpret each axis, even though it is mentioned in the text. For example, what does a large mcf or small mcf mean? What does a positive (red) or negative (blue) F() mean?*

   Our response: Thank you for your suggestion. We have modified Fig. 4 by adding the top panel showing mcf curves and Moran's I correlation in MHCII of 15 patients ($\approx$ 10%) with "high" survival (i.e., longest censored survival times) and 15 patients with "low" survival (i.e., shortest survival times), colored in black and red, respectively. The bottom panel of Fig. 4 now displays two estimated surface plots obtained by fitting two separate models using mcf curves and correlation in MHCII as functional covariates, respectively. Then, the legend for Fig. 4 has been edited as follows.

   "**Estimated surface from AFCM using NSCLC dataset.** (a) Mcf curves corresponding to "low" survival (i.e., shortest survival times) and "high" survival (i.e., longest observed censored times) patients in red and black, respectively. Note that mcf values below 1 indicate strong clustering of cells of same type, while values above 1 suggest higher level of mixing in of the two cell types. (b) Moran's I correlation using MHCII expression corresponding to "low" survival (i.e., shortest survival time) and "high" survival (i.e., longest observed censored time) patients in red and black, respectively. Moran's I values above 0 indicate a direct relationship in MHCII expression between tumor and stromal cells. Negative Moran's I values suggest an inverse association in MHCII expression between tumor and stromal cells. (c) Estimated surface from AFCM using mcf curves as functional covariates with values decreasing from positive (red) to negative (blue). (d) Estimated surface from AFCM using

Moran's I correlation in MHCII as functional covariates with values decreased from positive (red) to negative (blue). Positive $\hat{F}$ corresponds to increased risk of mortality while negative $\hat{F}$ associates with reduced hazard of death."

We have also added more details to the legends of other figures in the revised manuscript.

5. *Line 181 "In particular, if tumor cells clustered together in the neighborhood of 25 – 75μm, the overall survival improved, based on the negative estimate of the log hazard ratio. However, at distances beyond 75μm, the more tumor cells clustered together, the higher the risk of mortality, while the increased infiltration level of tumor-associated stromal cells reduced the hazard of death." A few points below:*

   (a) *This conclusion would be easier to make if the F() in Figure 4 was centered on white.*

   Our response: We have edited the estimated surface plots such that the color scale is transitioned from red (i.e., positive) to blue (i.e., negative), and centered on white (i.e., 0). Please see Figures 4 and 6 in our revised manuscript.

   (b) *I am not a fan of the terminology "clustered together". My preference would be something like, "if strong clustering of tumor cells is observed in the neighborhood of 25 - 75μm". Or clustering more than expected maybe?*

   Our response: We have changed the terminology "clustered together" to "strong clustering" and "clustered more than expected" throughout our revised manuscript.

   (c) *The difference in F() appears much larger for r > 100 than 254. Can you elaborate any more on the complete flipping of interpretation/association that happens around r = 90 in 4a and 4b? It is a hard concept to wrap my head around.*

   Our response: We are sorry about the confusion in the previous Figure 4a and 4b. Figure 4a displays the impact of mcf on the hazard ratio while holding other scalar covariates constant. On the other hand, Figure 4b illustrates the effect of correlation in MHCII between tumor and stromal cells on the hazard ratio. We have edited Figure 4 to aid the interpretation, such that the top panel includes mcf curves and Moran's I correlation in MHCII of 15 patients ($\approx 10\%$) with "high" survival (i.e., longest censored survival times) and 15 patients with "low" survival (i.e., shortest survival times), colored in black and red, respectively. The bottom panel of Figure 4 now displays two estimated surface plots obtained by fitting two separate models using mcf curves and correlation in MHCII as functional covariates, respectively.

(d) *As 4a and 4b are coloured by F(), would it also be useful for interpretation for 3b to be moved to Figure 4? Can the lines in Figure 3b also be coloured? Does that make any sense at all? Perhaps you could leave 3b in Figure 3, but then include it in Figure 4 with the top 10% "best survival" patients highlighted in red and bottom 10% "worst survival" patients highlighted in blue? Or some other highlighting that makes sense.*

Our response: Thank you, we think this is a great suggestion and have edited the Figures accordingly. Specifically, we have edited Figure 4 such that the top panel includes mcf curves and Moran's I correlation in MHCII of 15 patients ($\approx$ 10%) with "high" survival (i.e., longest censored survival times) and 15 patients with "low" survival (i.e., shortest survival times), colored in black and red, respectively. The bottom panel of Figure 4 now displays two estimated surface plots corresponding to mcf and correlation in MHCII, respectively.

6. *Figure 5: Why are 5c and 5d two plots? Wouldn't it make more sense for them to be one plot coloured by mixed/not mixed?*

   Our response: We have edited Figure 5 such that panel (c) now illustrates the mcf curves for the two categories "compartmentalized" vs. "mixed", colored in black and red, respectively.

7. *On line 246: "The authors concluded that P53 positivity associated with negative prognostic significance in breast cancer patients." "In particular, a sample was defined as P53 positive if any cancer cells was positively expressed." Pan et al's conclusions appear to be about P53 positive cancer cells. Can you explain what it means for P53 to be correlated between cancer and immune cells? Is this a problem with cell segmentation? Or does P53 have the same function in immune cells as it does cancer cells?*

   Our response: We have added the following paragraph to Section 3.1 of the revised manuscript to elaborate on the motivation for modelling the correlation in P53 expression between tumor and immune cells.

   "Inspired by the findings [by Pan et al.'s findings [1]], we investigated the distributions of P53 expression in tumor and immune cells separately for each patient. We observed that in P53 positive patients, the distribution of P53 intensity in immune cells was extremely right-skewed while that marker expression of tumor cells were distributed more symmetrically and centered at a much higher intensity, as shown in Fig S1 (a) in the Supporting Information. On the other hand, the P53 expression distributions were right-skewed, similarly between tumor

and immune cells in P53 negative patients (Fig S1 (b)). In other words, tumor and immune cells in P53 negative samples were more likely to be correlated while it might not be the case for P53 positive samples. This motivated us to explore the correlation in P53 between the two cell types as a function of cross-type cell distance (Fig. 6 (b)) without necessarily dichotomizing samples into "P53 positive" vs "P53 negative" groups, via Moran's I metric (Eq. 2). In a few patients, immune and tumor cells lying within 100 μm neighborhood radius, were positively correlated. As cell distance became larger, the correlation dropped close to 0. Though the correlation was rather weak, it was still worth studying the impact of the relationship between tumor and immune cells with respect to the P53 expression on patient survival using the proposed approach."

In other words, this is more of an exploratory analysis to demonstrate an alternative model via functional data analysis approach. Unfortunately, with limited sample size, there is no significant association between the tumor - immune P53 correlation and patient overall survival.

8. *The introduction could potentially benefit from some edits to make it easier and quicker to digest. For instance, in the paragraph starting on line 14, I would suggest spending more time talking about multiplexed imaging techniques and avoid detouring to mention bulk/sc sequencing without context.*

   Our response: Thank you for your suggestion. We have removed sentences describing conventional bulk/single cell sequencing techniques and added more information about multiplex tissue imaging in the second paragraph of the Introduction Section in the revised manuscript, as follows.

   "With advancements in technology, great progress has been made in high parameter imaging of tissues in situ to allow simultaneous quantification and visualization of individual cells in tissue sections. More specifically, multiplex tissue imaging (MTI) [2] methods such as cyclic immunoflourescence (CyCIF) [3], CO-Dectection by indEXing (CODEX) [4], multiplex immunohistochemistry (mIHC) [5], imaging mass cytometry (IMC) [6], and multiplex ion beam imaging (MIBI) [7] are capable of measuring the expression of tens of markers at single-cell resolution while preserving the spatial distribution of cells. As an example, multiplex immunohistochemistry (mIHC) detects and visualizes specific antigens in cells of a tissue section by utilizing antibody-antigen reactions coupled to a flourescent dye or an enzyme [8, 9].

Another instance includes multiplexed ion beam imaging (MIBI) [7], which utilizes secondary ion mass spectrometry to image metal-conjugated antibodies. As such, MIBI enables single-cell analysis of up to 100 parameters without spectral overlap between channels. Altogether, imaging provides an additional dimension of spatial resolution to the single cell signature profiles, which in turn allows researchers not only to study cellular composition but also to make inferences about specific cell-cell interactions."

9. *This approach clearly extends beyond studying the TME, I'm not sure how or if it is worth emphasizing this?*

   Our response: Thank you, we agree that this approach has broad applicability beyond the TME. We have added the following paragraph to the Conclusions and Discussion section of the revised manuscript to discuss possible generalizations of our approach.

   "Motivated by our available datasets, we specifically focus on studying the TME and how spatial heterogeneity of cells within the TME impacts patient overall survival in this article. However, the proposed framework could certainly be extended to other analyses in two directions. First, the spatial architecture of cells being investigated is not necessarily restricted to the TME. Second, other types of response variables other than survival outcomes could be modelled, with necessary modifications made to the model parameterization and estimation procedure indeed. We take the study of human endocrine pancreas and immune system in type 1 diabetes (T1D) using images of pancreatic tissue sections collected from imaging mass cytometry (IMC) platform by Wang et al. [10] as an example. With abundance and localization of proteins at single-cell resolution available, the spatial interaction of immune and pancreatic epithelial cells in pancreatic islets could be captured by the mark connection functions (mcf). Furthermore, with the donors in the dataset being categorized into "controls" (i.e., no history of diabetes) and "T1D" groups, one could use a logistic regression model with the resulting mcfs between immune and pancreatic epithelial cells served as functional covariates, to make inferences on how the islet spatial architecture differs between the two groups and to gain insights into the progression of T1D. By switching to such outcomes from the exponential family, our proposed model would essentially become a functional generalized additive model, which McLean et al. [11] and Muller et al. [12] have discussed in detail."

10. *The authors don't appear to refer to p-values / statistical significance at any point. Are the "Estimates from AFCM plots" purely for qualitative interpretation? If I were to use your*

method for my own analysis, is there a single value I can report to provide evidence of a relationship? How would someone use these results?

Our response: We have added a summary output of the model for the NSCLC dataset in Sections S.1.1 and S.1.2 of the Supporting Information, in which the approximated significance of the smooth term is provided. Additionally, the following paragraph has been included in Section 3.1 of the revised manuscript.

"Section S1.1.1 in the Supporting Information reported the summary output of the corresponding model. The p-value of 0.44 indicated no significant nonlinear association between the spatial interactions of tumor and stromal cells with overall survival. However, note that given a relatively large number of parameters to be estimated, our sample size might not provide enough power to detect such association. As a result, the qualitative interpretation of the model using the surface plots is still worthwhile."

11. *The authors make reference to multiplex data which can quantify many cell types simultaneously however their examples only compare two types of cells (cancer vs immune). The authors briefly mention expanding the tests to include multiple or correlated objects in the conclusion. However, have the authors considered how they would simply apply this approach to multiple pairwise cell type comparisons? Or testing many markers for these comparisons? Is there a sensible way to rank pairwise comparisons? Or would I need to look at all pairwise plots and make a qualitative judgement?*

    Our response: Thank you for your great insights. By utilizing the spatial summary functions discussed in the paper, we could only apply the proposed framework in a pairwise fashion, i.e., two cell types at a time (tumor vs. stromal, tumor vs. immune, tumor vs. CD4+ T cells, etc.). We totally agree with you that we could extend the approach to model all possible pairs of cell types, and then adjust for multiple comparisons to reach the conclusions. However, we also think that leaving the judgement call to the investigators regarding which pair of cell types being modelled based on their research question is a legitimate approach.

12. *Overall, I found the manuscript easy to read. However, I will leave it to the editors to comment on the structure as it does not follow the "manuscript organisation" outlined on PLOS Computational Biology's website. Some components of the text could be moved from the results into the methods section and the discussion (conclusion) section.*

Our response: We thank you again for your constructive comments. We have restructured the paper by moving the paragraphs describing the two datasets (NSCLC and TNBC) to the Materials and Methods Section.

Responses to Reviewer 2

We sincerely thank you for providing us constructive suggestions, which led to a significant improvement of the paper. We now provide point to point responses to your comments. For your convenience, we reiterate your comments in italics before responding.

1. *This study aim to illustrate the use of established spatial statistics in studying cellular interactions. They motivate their work by discussing the role of tumour microenvironment in cancer evolution. They explore several spatial statistics in different datasets. In general, the concepts presented in the paper are interesting. However, the presented results show that these metrics are not really very useful in reflecting the biology. For example: "if tumor cells clustered together in the neighborhood of 25 – 75μm, the overall survival improved, based on the negative estimate of the log hazard ratio. However, at distances beyond 75μm, the more tumor cells clustered together, the higher the risk of mortality". This does not make sense as increased cancer cell density should reflect a worse outcome. Similarly, other statements regard the other analyses is again difficult to interpret. This applies to other statements in the paper.*

   Our response: We appreciate your input. We agree with you that increased cancer cell density usually reflects a higher hazard of mortality. However, the density measure alone does not take into account any spatial interactions between tumor cells and other cells in the tumor microenvironment. Though the spatial statistics utilized in this paper has been established, the novelty of our approach is the combination of these established statistics with functional data analysis to flexibly model the cell-cell interactions at different distances. Additionally, the proposed model provides an interpretation of the nonlinear relationship between spatial patterns of cells and overall survival.

   Regarding the interpretation you point out, it is more related to tumor and stromal cells being clustered in their own compartments rather than mixing together in the span of 25–75 μm radius. This is in line with Keren et al.'s [13] findings that spatial separation between tumor and immune cells improves survival. However, if such behavior continues beyond 75 μm, the more tumor cells cluster, the higher the risk of mortality. In general, these interpretations are dependent on the distances between cells.

2. *Except for the introduction, the paper is very hard to follow. It reads as disparate pieces of experiments that are put together and no clear link between the sections.*

Our response: Thank you for your input. We have restructured the paper by moving the paragraphs describing the two datasets (NSCLC and TNBC) to the end of the Materials and Methods Section, and included the following paragraph at the beginning of Section 2 to hopefully improve the transition of the paper.

"The main goal of our proposed approach is to model the spatial heterogeneity of cells in histological images and patient overall survival in a functional data analysis framework, in addition to other scalar clinical variables. As such, summary functions capturing the spatial architecture of cells in each image are necessary inputs of our model. We utilize mark connection function (mcf) [14] and Moran's I statistic [15] to represent qualitative and quantitative spatial information, respectively. We then model the available spatial input functions using the Additive Functional Cox Model. Details are given below."

3. *Showing few survival values independent of any other information (e.g Fig. 3C) does not add any information.*

   Our response: Thank you for your suggestion. We have removed survival time plots (i.e., Figures 3c and 6c in previous version) in our revised manuscript.

4. *It is not clear how surface plots can be useful.*

   Our response: We are interested in studying the impact of spatial interactions between cells on survival outcomes in conjunction with other scalar predictors such as age and disease stage, via functional modelling approach. After the estimation process, we use surface plots to illustrate the predicted hazard of mortality at different possible combinations of cell distances and functional values (i.e., mcf or Moran's I correlation as seen in Section 3) while fixing the scalar variables constant. In other words, we utilize the surface plots to interpret the impact of the spatial interactions of cells on survival outcomes.

   We agree that our original manuscript did not have sufficient explanation of the utility of these figures. To improve upon this, we have greatly expanded the legends of Figures 4 and 6 in our revised manuscript to aid interpretation of the surface plots.

5. *It was not clear why simulation was used at the end.*

   Our response: We use simulation studies to show that the proposed method produces reasonable results in line with how we expect it to perform. By mimicking the characteristics of the real data (i.e., NSCLC dataset), we assume that clinical scalar variables (e.g., age,

disease stage, etc.) and spatial interactions of cells in tissue samples both affect the observed survival times. The cell interaction patterns are functions of inter-cell distances. In other words, we build a true survival model which involves both scalar and functional terms as in Eq. (7), with their corresponding contributions to the model specified via scalar coefficient β and the true functional form F. We then evaluate the performances of three different models: (1) our proposed model accounting for both functional and scalar effects, (2) model accounting for only functional term, and (3) model accounting for only scalar term, at four different sample sizes. Our goal is to demonstrate that if the observed survival times were in fact generated from the specified process which involved both scalar and functional effects, failing to account for either could lead to a decrease in model predictive power.

6. *It was not clear why only the centres of the cells were shown and no original cell images.*
   Our response: We appreciate your comment. Our interest lies in modelling the association between spatial interactions of cells and survival outcomes, in additional to clinical predictors. As a result, only relative distances between cells, which are calculated directly from the centroid of each cell, are sufficient for our purpose. We use the scatter plots with coordinates of cell centers just to visualize the spatial distributions of cell types of interest. Original cell images for the NSCLC and TNBC datasets can be found in Johnson et al. [16] and Keren et al. [13], respectively.

References

[1] Pan Y, Yuan Y, Liu G, Wei Y. P53 and Ki-67 as prognostic markers in triple-negative breast cancer patients. PLoS One. 2017;12(2):e0172324.

[2] Burlingame EA, Eng J, Thibault G, Chin K, Gray JW, Chang YH. Toward reproducible, scalable, and robust data analysis across multiplex tissue imaging platforms. Cell reports methods. 2021;1(4):100053.

[3] Lin JR, Izar B, Wang S, Yapp C, Mei S, Shah PM, et al. Highly multiplexed immunofluorescence imaging of human tissues and tumors using t-CyCIF and conventional optical microscopes. Elife. 2018;7.

[4] Goltsev Y, Samusik N, Kennedy-Darling J, Bhate S, Hale M, Vazquez G, et al. Deep profiling of mouse splenic architecture with CODEX multiplexed imaging. Cell. 2018;174(4):968–981.

[5] Tsujikawa T, Kumar S, Borkar RN, Azimi V, Thibault G, Chang YH, et al. Quantitative multiplex immunohistochemistry reveals myeloid-inflamed tumor-immune complexity associated with poor prognosis. Cell reports. 2017;19(1):203–217.

[6] Giesen C, Wang HA, Schapiro D, Zivanovic N, Jacobs A, Hattendorf B, et al. Highly multiplexed imaging of tumor tissues with subcellular resolution by mass cytometry. Nature methods. 2014;11(4):417–422.

[7] Angelo M, Bendall SC, Finck R, Hale MB, Hitzman C, Borowsky AD, et al. Multiplexed ion beam imaging of human breast tumors. Nature medicine. 2014;20(4):436–442.

[8] Maiques O, Georgouli M, Sanz-Moreno V. Recent advances in tissue imaging for cancer research. F1000Research. 2019;8.

[9] Duraiyan J, Govindarajan R, Kaliyappan K, Palanisamy M. Applications of immunohistochemistry. Journal of pharmacy & bioallied sciences. 2012;4(Suppl 2):S307.

[10] Wang YJ, Traum D, Schug J, Gao L, Liu C, Atkinson MA, et al. Multiplexed in situ imaging mass cytometry analysis of the human endocrine pancreas and immune system in type 1 diabetes. Cell metabolism. 2019;29(3):769–783.

[11] McLean MW, Hooker G, Staicu AM, Scheipl F, Ruppert D. Functional generalized additive models. Journal of Computational and Graphical Statistics. 2014;23(1):249–269.

[12] Müller HG, Wu Y, Yao F. Continuously additive models for nonlinear functional regression. Biometrika. 2013;100(3):607–622.

[13] Keren L, Bosse M, Marquez D, Angoshtari R, Jain S, Varma S, et al. A structured tumor-immune microenvironment in triple negative breast cancer revealed by multiplexed ion beam imaging. Cell. 2018;174(6):1373–1387.

[14] Baddeley A, Rubak E, Turner R. Spatial point patterns: methodology and applications with R. CRC press; 2015.

[15] Moran PA. The interpretation of statistical maps. Journal of the Royal Statistical Society: Series B (Methodological). 1948;10(2):243–251.

[16] Johnson AM, Boland JM, Wrobel J, Klezcko EK, Weiser-Evans M, Hopp K, et al. Cancer cell-specific MHCII expression as a determinant of the immune infiltrate organization and function in the non-small cell lung cancer tumor microenvironment. Journal of Thoracic Oncology. 2021;.
