# Supplementary material for: SPF: A spatial and functional data analytic approach to cell imaging data
Source: PLoS Comput Biol. 2022 Jun 15;18(6):e1009486. doi: 10.1371/journal.pcbi.1009486 (PMC9239468; doi:10.1371/journal.pcbi.1009486)
Supplement: Attachment — Submitted filename: R2_Letter.pdf [file pcbi.1009486.s009.pdf]

Dear Dr. Meier-Schellersheim and Dr. Haugh,

Thank you very much for reviewing our manuscript entitled: “SPF: A Spatial and Functional Data Analytic Approach to cell Imaging data” (PCOMPBIOL-D-21-01681R1). We are pleased to submit our revised manuscript for publication in PLOS Computational Biology. Two versions of the revised paper are included: one with changes highlighted in yellow and a clean version.

We really appreciate the reviewers’ providing us constructive comments and suggestions, which led to a significant improvement of our paper. To facilitate the review process, in our letter, we provide a point-by-point response to each of the comments and mention the changes made to the original manuscript. We summarize the major changes below:

- Figure 1 has been replaced with another set of images to illustrate the utility of the mark connection function more effectively. In addition, we have modified Figure 2 with increased font size for the axis labels and added a vertical line at $r = 10$ μm.
- In Section 2.3, we have reported the algorithms used for cell segmentation in the NSCLC and TNBC datasets.
- In the Discussion Section, we have elaborated on the limitations of the proposed framework in terms of model complexity and result interpretation. We have also discussed potential issues with processing TMA images collected from Vectra and possible approaches to address them.

Thank you very much for your time and consideration. We look forward to hearing from you.

Sincerely,

Thao Vu, Ph.D.

Responses to Reviewer 2

We sincerely thank you for providing us constructive suggestions, which led to a significant improvement of the paper. We now provide point to point responses to your comments. For your convenience, we reiterate your comments in italics before responding.

1. *The proposed metrics can reveal interesting insights, however these are not always easy to interpret. It will be useful to discuss the limitations in the discussion.*

   Our response: Thank you. In response to your suggestion, we have added the following paragraph to the Discussion section of the revised paper.

   "Though the additive model framework provides us flexibility to model nonlinear relationship between spatial cell distribution and overall survival, there exist a few limitations. Such complex model requires a relatively large number of parameters to be estimated, which compromises our power to detect significant results provided our limited sample size, as we discuss in Sections 3.1 and 3.2. Recall that the study by Cui et al. [1] demonstrating the application of AFCM in quantifying the association between physical activity and survival data included 2816 participants. Additionally, as with other nonlinear models, the interpretation of results might not always be easy for all applications. We try to draw the connection between our conclusions and Johnson et al.'s that increased immune - tumor cell interactions beyond 100 μm reduces risk of mortality (Section 3.1). For inter-cell distances in the span of 25–75 μm radius, on the other hand, our results indicate a different implication that the more tumor cells cluster, the lower the hazard of death. Note that we have specifically chosen mark connection functions (mcf) and Moran's I correlation as representative metrics to summarize qualitative and quantitative spatial information, respectively, in this paper. Other summary functions could be employed to capture various levels of spatial information, depending on the applications. For instance, with the interest in the level of immune infiltration, Barua et al. [2] utilized the G-cross function to quantify the distribution of the nearest immune cells relative to tumor cells within any given inter-cell distance. In such instance, the observed G-cross function could then be used in place of the mcf as functional covariates in the proposed approach. "

2. *The authors did not report on the methods they used for image analysis which only appeared in their response to reviewer 1. Multiplexed images can be extremely challenging and reasonable*

segmentation accuracy is needed for reliable conclusions. Details of the image analysis and results (raw images and segmented images) should be included. I appreciate the paper is presenting a method for studying the resulting spatial single cell distribution. However, making it clear in the discussion of potential errors and challenges in data segmentation should be taken into consideration when interpreting the results.

Our response: We agree with you that the results of any downstream analyses rely heavily on the preprocessed data. In our case, potential errors posed in the cell segmentation step could certainly affect the spatial analysis results. Following your suggestion, we have added this sentence: "The acquired images were then processed using Akoya's inForm tissue analysis software to obtain a data matrix with rows corresponding to individual cells and columns corresponding to x- and y-coordinates of each cell on the image, individual marker expression, and cell phenotypes" to the NSCLC dataset in Section 2.3. Similarly, this sentence: "Cell segmentation was performed by adapting a convolutional neural network (CNN) approach, i.e., DeepCell [3] for nuclear segmentation to MIBI data. Details of the method were described in Keren et al. [4]." has been added to the TNBC dataset in Section 2.3.

Finally, we have added the following paragraph to the Discussion section of the revised manuscript.

"Similar to other spatial analyses, our proposed approach relies on the cell-segmented data. In other words, if cells are not segmented correctly from raw images, the resulting spatial distribution of cells might not accurately reflect the underlying tissue architecture. Accordingly, results of any downstream analyses associating spatial summary information with a clinical outcome of interest would certainly be impacted. As we briefly discuss in Section 2.3, Johnson et al. [5] utilized the state-of-the-art commercial software, inForm, for cell and nucleus segmentation for the NSCLC dataset, while Keren et al. [4] adapted a convolutional neural network approach, DeepCell [3], to extract segmented data in the TNBC dataset. While it is possible that errors could potentially occur with either segmentation approach, this has not thoroughly been investigated and is beyond the scope of our current analysis."

3. *Following from that point, the red data points that in Figure 1 for Patient 131 are expected to be wrongly segmented cells as TMAs are usually cut as a round biopsy. These should be excluded from the data through QC. Could the author confirm this from the raw images or replace*

Our response: We agree with you that the image for Patient 131 was poorly chosen as an example to illustrate that the differences in spatial patterns of cells would be reflected through mcf curves. We have now replaced Figure 1 in the revised manuscript with another set of images from the non-small cell lung cancer (NSCLC) dataset.

Recall that the ovarian cancer dataset in our paper were collected by staining and imaging tissue microarray (TMA) slides, as reported in detail by Jordan et al. [6]. We have added the following paragraph to the Discussion Section of the revised paper.

"After this paper had been submitted, we discovered a study by Wilson et al. [7], which discussed issues with imaging data collected from tissue microarrays (TMA). In particular, the authors highlighted the plausibility of the tissue slides being folded or torn during the slicing process, leading to some sections of the image with no cells present (e.g., Fig. S4 (c) of Supporting Information). To overcome such challenge, Wilson et al. [7] introduced a framework (with the accompanying `R` package `SpatialTIME` [8]) that utilized the K-function in a permutation-based setting to account for such drawbacks. Inspired by the study, it would be interesting to extend our proposed framework to include the permutation-based summary function. More specifically, we would randomly permutate the cell labels to obtain the empirical distribution of the mcfs. Then, the mean mcf instead of the observed counterpart would then be used as functional covariate in the additive functional Cox model (AFCM) to investigate the association between spatial heterogeneity of cells and patient overall survival."

4. *That AFCM does not result in any statistically significant signatures when using this metric, raises a concern regarding their utility of these metrics which make the study merely exploratory. Could the authors elaborate on the potential reason in the discussion?*

   Our response: Thank you. We agree with you that we focus more on the exploratory analyses in this paper due to lack of power in detecting significant results. We have added the following paragraph in the Discussion Section of the revised manuscript.

   "Though the additive model framework provides us flexibility to model nonlinear relationship between spatial cell distribution and overall survival, there exist a few limitations. Such complex model requires a relatively large number of parameters to be estimated, which compromises our power to detect significant results given our limited sample size, as we discuss in Sections 3.1 and 3.2. Recall that the study by Cui et al. [1] demonstrating the application of AFCM in quantifying the association between physical activity and survival data

included 2816 participants. Additionally, as with other nonlinear models, the interpretation of results might not always be easy for all applications. We try to draw the connection between our conclusions and Johnson et al.'s that increased immune - tumor cell interactions beyond 100 μm reduces risk of mortality (Section 3.1). For inter-cell distances in the span of 25–75 μm radius, on the other hand, our results indicate a different implication that the more tumor cells cluster, the lower the hazard of death. Note that we have specifically chosen mark connection functions (mcf) and Moran's I correlation as representative metrics to summarize qualitative and quantitative spatial information, respectively, in this paper. Other summary functions could be employed to capture various levels of spatial information, depending on the applications. For instance, with the interest in the level of immune infiltration, Barua et al. [2] utilized the G-cross function to quantify the distribution of the nearest immune cells relative to tumor cells within any given inter-cell distance. In such instance, the observed G-cross function could then be used in place of the mcf as functional covariates in the proposed approach. "

5. *Fig. 2: The text is difficult to read The authors refer to* $r = 10$ *in the text, it will be helpful to add a vertical line to indicate this on the figure*

   Our response: Thank you. We have modified Figure 2 in the revised manuscript with increased font size for the axis labels and added a vertical line at $r = 10$ μm.

6. *There are few mistakes in referring to the supplementary material: e.g. Line 185 should refer to Fig S2 not S1 and line 283 should refer to Fig. S3 not S1. Line 298 should refer to Section 3 not 2.*

   Our response: Thank you. We have corrected all the figure/section misreferences in the revised manuscript.

7. *I am not sure why the ovarian dataset and the associated analysis was moved to the supplementary. It should be fit in the appropriate sections of the study.*

   Our response: Thank you. We initially wanted to demonstrate the applicability of the proposed framework using data collected from two different imaging platforms: Vectra and MIBI. Since the ovarian and NSCLC datasets were both collected from Vectra, we decided to present the NSCLC analysis in the main text since it had a slightly larger sample size compared to that of the ovarian dataset (153 vs. 114). In addition, our current analysis

has not yet thoroughly investigated potential issues with TMA images in the ovarian dataset (please see our response to your comment #3), which we recently discovered after submitting the manuscript. Therefore, it would be more appropriate for us to still keep the analysis of the ovarian dataset in the Supporting Information.

References

[1] Cui E, Crainiceanu CM, Leroux A. Additive Functional Cox Model. Journal of Computational and Graphical Statistics. 2020; p. 1–14.

[2] Barua S, Fang P, Sharma A, Fujimoto J, Wistuba I, Rao AU, et al. Spatial interaction of tumor cells and regulatory T cells correlates with survival in non-small cell lung cancer. Lung Cancer. 2018;117:73–79.

[3] Van Valen DA, Kudo T, Lane KM, Macklin DN, Quach NT, DeFelice MM, et al. Deep learning automates the quantitative analysis of individual cells in live-cell imaging experiments. PLoS computational biology. 2016;12(11):e1005177.

[4] Keren L, Bosse M, Marquez D, Angoshtari R, Jain S, Varma S, et al. A structured tumor-immune microenvironment in triple negative breast cancer revealed by multiplexed ion beam imaging. Cell. 2018;174(6):1373–1387.

[5] Johnson AM, Boland JM, Wrobel J, Klezcko EK, Weiser-Evans M, Hopp K, et al. Cancer cell-specific MHCII expression as a determinant of the immune infiltrate organization and function in the non-small cell lung cancer tumor microenvironment. Journal of Thoracic Oncology. 2021;.

[6] Jordan KR, Sikora MJ, Slansky JE, Minic A, Richer JK, Moroney MR, et al. The capacity of the ovarian cancer tumor microenvironment to integrate inflammation signaling conveys a shorter disease-free interval. Clinical Cancer Research. 2020;26(23):6362–6373.

[7] Wilson CM, Thapa R, Creed J, Nguyen J, Segura CM, Schildkraut J, et al. Statistical framework for studying the spatial architecture of the tumor immune microenvironment. medRxiv. 2021;.

[8] Creed JH, Wilson CM, Soupir AC, Colin-Leitzinger CM, Kimmel GJ, Ospina OE, et al. spatialTIME and iTIME: R package and Shiny application for visualization and analysis of immunofluorescence data. Bioinformatics. 2021;37(23):4584–4586.
